# Dichlorido[methyl 2-(quinolin-8-yl­oxy-κ^2^ *N*,*O*)acetate-κ*O*]mercury(II)

**DOI:** 10.1107/S1600536812026591

**Published:** 2012-06-20

**Authors:** Yu-Hong Wang, Xue-Hua Zhu, Rui-Feng Song

**Affiliations:** a School of Chemistry and Bioengineering, Suzhou University of Science and Technology, Suzhou 215009, People’s Republic of China

## Abstract

In the neutral title complex, [HgCl_2_(C_12_H_11_NO_3_)], the Hg^II^ ion is penta­coordinated by two Cl atoms, one N atom and two weakly coordinating O atoms from the methyl 2-(quinolin-8-yl­oxy)acetate ligand. The coordination around the Hg^II^ ion may be described as highly distorted trigonal–bipyramidal. Centrosymmetric dimers are formed by an additional weak Hg⋯Cl inter­action, leading to a distorted octa­hedral coordination geometry around the Hg^II^ ion.

## Related literature

For the use of quinolin-8-yl­oxy acetic acid and its derivatives as ligands in transition metal complexes, see: Cheng *et al.* (2007 ▶); Song *et al.* (2004 ▶); Wang *et al.* (2005 ▶, 2008 ▶). 
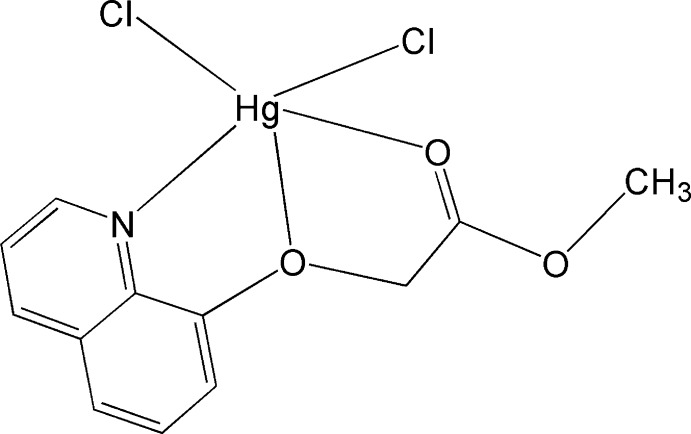


## Experimental

### 

#### Crystal data

[HgCl_2_(C_12_H_11_NO_3_)]
*M* *_r_* = 488.71
Triclinic, 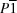

*a* = 7.2644 (4) Å
*b* = 9.7607 (2) Å
*c* = 10.8411 (6) Å
α = 71.317 (7)°
β = 75.453 (7)°
γ = 69.816 (8)°
*V* = 674.87 (5) Å^3^
*Z* = 2
Mo *K*α radiation
μ = 11.80 mm^−1^
*T* = 223 K
0.50 × 0.25 × 0.10 mm

#### Data collection

Rigaku Saturn diffractometer
Absorption correction: multi-scan (*REQAB*; Jacobson, 1998 ▶) *T*_min_ = 0.067, *T*_max_ = 0.385
5090 measured reflections
2432 independent reflections
2330 reflections with *I* > 2σ(*I*)
*R*_int_ = 0.050

#### Refinement

*R*[*F* ^2^ > 2σ(*F* ^2^)] = 0.052
*wR*(*F* ^2^) = 0.142
*S* = 1.07
2432 reflections
174 parameters
H-atom parameters constrained
Δρ_max_ = 3.58 e Å^−3^
Δρ_min_ = −2.39 e Å^−3^

### 

Data collection: *CrystalClear* (Rigaku/MSC, 2001 ▶); cell refinement: *CrystalClear*; data reduction: *CrystalStructure* (Rigaku/MSC, 2004 ▶); program(s) used to solve structure: *SHELXS97* (Sheldrick, 2008 ▶); program(s) used to refine structure: *SHELXL97* (Sheldrick, 2008 ▶); molecular graphics: *SHELXTL* (Sheldrick, 2008 ▶); software used to prepare material for publication: *SHELXTL*.

## Supplementary Material

Crystal structure: contains datablock(s) I, global. DOI: 10.1107/S1600536812026591/im2385sup1.cif

Structure factors: contains datablock(s) I. DOI: 10.1107/S1600536812026591/im2385Isup2.hkl

Additional supplementary materials:  crystallographic information; 3D view; checkCIF report

## Figures and Tables

**Table 1:** Selected bond lengths (Å)

Hg1—Cl1	2.340 (2)
Hg1—Cl2	2.350 (2)
Hg1—N1	2.463 (6)
Hg1—O1	2.746 (6)
Hg1—O3	2.876 (6)
Hg1—Cl1^i^	3.204 (2)

Symmetry code: (i) 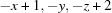.

**Fig. 1.**
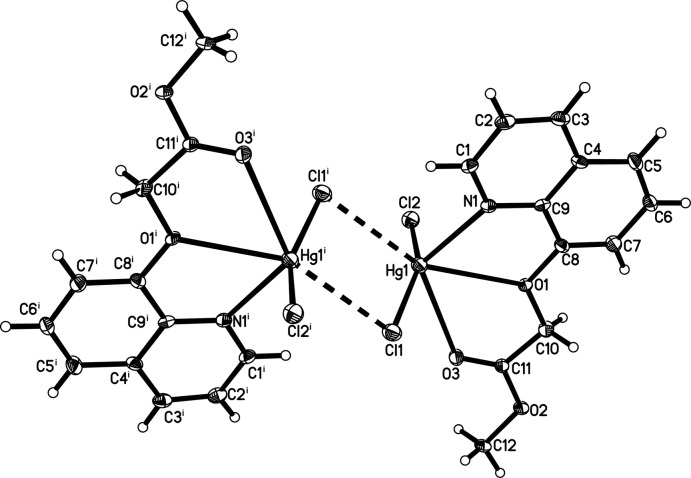
The molecular structure of (I), with 30% probability displacement ellipsoids [symmetry codes: (i) 1 - x, -y, 2 - z]. The dashed line indicates the weak Hg···Cl interaction.

**Fig. 2.**
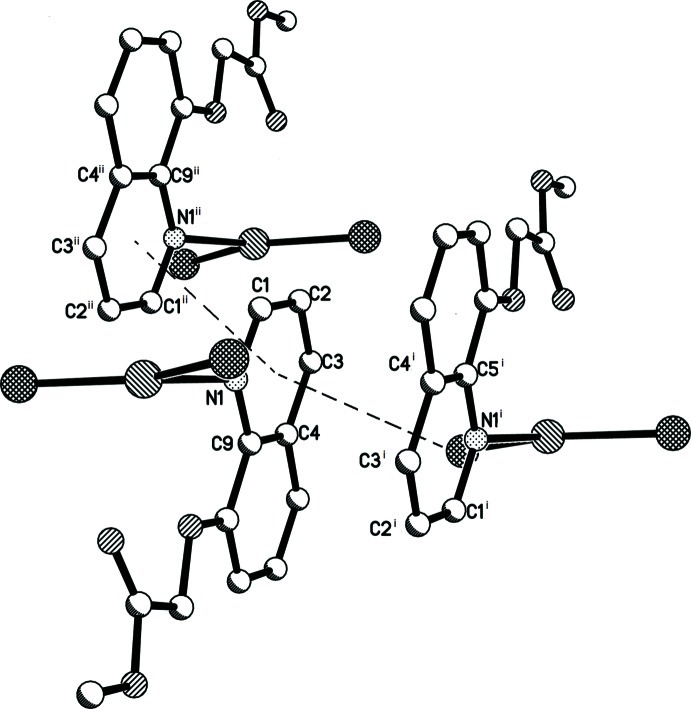
A view of intermolecular π-π interactions, interactions between the parallel quinoline rings of neighbouring complexes [symmetry codes: (i) 1 - x, 1 - y, 2 - z; (ii) -x, 1 - y, 2 - z].

## supplementary crystallographic information

### Comment

Quinolin-8-yloxy acetic acid and it's derivatives are well known ligands in transition metal coordination compounds (Cheng *et al.*, 2007; Song *et al.*, 2004; Wang *et al.*, 2005; Wang *et al.*, 2008). Some metal complexes with such ligands are being prepared because of their intriguing structural diversity and potential uses as functional materials (Cheng *et al.*, 2007; Song *et al.*, 2004; Wang *et al.*, 2005; Wang *et al.*, 2008). So, we prepared the title Hg^II^ complex with methyl-2-(quinoline-8-yloxy)-acetate ligand, (I).

In the title compound, the Hg^II^ atom is five-coordinated by two Cl atoms, one N atom and two O atoms from methyl-2-(quinoline-8-yloxy)-acetate ligand, forming a highly distorted trigonal bipyramidal geometry (Fig. 1). Hg—Cl bond lengths are 2.340 (2) and 2.350 (2) Å, and Hg—N bond lengths are 2.463 (6) Å. The weak coordinative Hg—O bond lengths are 2.746 (6) Å and 2.876 (6) Å. Angles around Hg are in a range of 56.55 (16)–153.41 (8)° (Table 1). If these are considered to be chemically signifcant interactions, two monoclear Hg complexes are formed into the centrosymmetric dimers by weak Hg—Cl interactions (Fig. 1). So, the coordination around Hg atom can act as a distrorted octahedral geometry.

The molecular packing is controlled by these dimers and intermolecular π-π interactions; the quinoline rings are separated by 3.527 (1) and 3.813 (1) Å (Fig. 2).

### Experimental

Quinolin-8-yloxy acetic acid (0.0203 g, 0.1 mmol), HgCl_2_ (0.0272 g, 0.1 mmol), methanol (3 ml) and triethylamine (0.0101 g, 0.1 mmol) were placed in a thick Pyrex tube and heated to 130 C° for 5 days. After cooling at a rate of 5 C°/h to ambient temperature, yellow prismatic crystals were collected, washed with anhydrous ethanol, and dried at room temperature. The yield is 51% based on quinolin-8-yloxy acetic acid. Analysis found: C, 29.91; H, 2.30; N, 2.87%; calculated for C_12_H_11_Cl_2_HgNO_3_: C, 29.49; H, 2.27; N, 2.87%.

### Refinement

H atoms were included in calculated positions and refined as riding, with C—H distances of 0.94 (aromatic), 0.98 (methylene) and 0.97 Å (methyl), and with *U*_iso_(aromatic and ethyl) = 1.2*U*_eq_(C) and *U*_iso_(methylene) = 1.5*U*_eq_(C).

### Crystal data

**Table:**

[HgCl_2_(C_12_H_11_NO_3_)]	*Z* = 2
*M**_r_* = 488.71	*F*(000) = 456
Triclinic, *P*1	*D*_x_ = 2.405 Mg m^−^^3^
Hall symbol: -P 1	Mo *K*α radiation, λ = 0.71075 Å
*a* = 7.2644 (4) Å	Cell parameters from 3438 reflections
*b* = 9.7607 (2) Å	θ = 3.0–27.5°
*c* = 10.8411 (6) Å	µ = 11.80 mm^−^^1^
α = 71.317 (7)°	*T* = 223 K
β = 75.453 (7)°	Prism, yellow
γ = 69.816 (8)°	0.50 × 0.25 × 0.10 mm
*V* = 674.87 (5) Å^3^	

### Data collection

**Table:**

Rigaku Saturn diffractometer	2432 independent reflections
Radiation source: fine-focus sealed tube	2330 reflections with *I* > 2σ(*I*)
Graphite monochromator	*R*_int_ = 0.050
Detector resolution: 14.63 pixels mm^-1^	θ_max_ = 25.5°, θ_min_ = 3.0°
ω scans	*h* = −8→8
Absorption correction: multi-scan (REQAB; Jacobson, 1998)	*k* = −11→9
*T*_min_ = 0.067, *T*_max_ = 0.385	*l* = −13→10
5090 measured reflections	

### Refinement

**Table:**

Refinement on *F*^2^	Primary atom site location: structure-invariant direct methods
Least-squares matrix: full	Secondary atom site location: difference Fourier map
*R*[*F*^2^ > 2σ(*F*^2^)] = 0.052	Hydrogen site location: inferred from neighbouring sites
*wR*(*F*^2^) = 0.142	H-atom parameters constrained
*S* = 1.07	*w* = 1/[σ^2^(*F*_o_^2^) + (0.114*P*)^2^] where *P* = (*F*_o_^2^ + 2*F*_c_^2^)/3
2432 reflections	(Δ/σ)_max_ = 0.001
174 parameters	Δρ_max_ = 3.58 e Å^−^^3^
0 restraints	Δρ_min_ = −2.39 e Å^−^^3^

### Special details

**Table:**

Geometry. All esds (except the esd in the dihedral angle between two l.s. planes) are estimated using the full covariance matrix. The cell esds are taken into account individually in the estimation of esds in distances, angles and torsion angles; correlations between esds in cell parameters are only used when they are defined by crystal symmetry. An approximate (isotropic) treatment of cell esds is used for estimating esds involving l.s. planes.
Refinement. Refinement of F^2^ against ALL reflections. The weighted R-factor wR and goodness of fit S are based on F^2^, conventional R-factors R are based on F, with F set to zero for negative F^2^. The threshold expression of F^2^ > 2sigma(F^2^) is used only for calculating R-factors(gt) etc. and is not relevant to the choice of reflections for refinement. R-factors based on F^2^ are statistically about twice as large as those based on F, and R- factors based on ALL data will be even larger.

### Fractional atomic coordinates and isotropic or equivalent isotropic displacement parameters (Å^2^)

**Table:**

	*x*	*y*	*z*	*U*_iso_*/*U*_eq_	
Hg1	0.48080 (4)	0.17195 (3)	0.82524 (2)	0.0283 (2)	
Cl1	0.7584 (4)	0.0079 (3)	0.9214 (2)	0.0343 (5)	
Cl2	0.2251 (4)	0.2330 (3)	0.7032 (2)	0.0328 (5)	
O1	0.5887 (9)	0.4276 (6)	0.6794 (5)	0.0267 (12)	
O2	0.9795 (10)	0.3004 (7)	0.4386 (6)	0.0293 (13)	
O3	0.7782 (11)	0.1803 (7)	0.5930 (6)	0.0350 (15)	
N1	0.3624 (11)	0.3914 (8)	0.9197 (6)	0.0229 (14)	
C1	0.2572 (13)	0.3719 (9)	1.0411 (8)	0.0250 (17)	
H1	0.2485	0.2744	1.0883	0.030*	
C2	0.1591 (14)	0.4918 (11)	1.1008 (8)	0.0291 (19)	
H2	0.0929	0.4727	1.1882	0.035*	
C3	0.1596 (13)	0.6348 (10)	1.0327 (8)	0.0280 (18)	
H3	0.0892	0.7160	1.0707	0.034*	
C4	0.2680 (12)	0.6597 (9)	0.9034 (7)	0.0229 (16)	
C5	0.2793 (15)	0.8066 (10)	0.8262 (9)	0.032 (2)	
H5	0.2102	0.8915	0.8595	0.038*	
C6	0.3906 (15)	0.8224 (9)	0.7043 (9)	0.0306 (19)	
H6	0.3995	0.9189	0.6541	0.037*	
C7	0.4920 (14)	0.6985 (10)	0.6522 (8)	0.0288 (19)	
H7	0.5649	0.7133	0.5665	0.035*	
C8	0.4873 (13)	0.5564 (9)	0.7230 (8)	0.0232 (16)	
C9	0.3727 (12)	0.5317 (9)	0.8506 (7)	0.0226 (16)	
C10	0.7341 (16)	0.4428 (10)	0.5679 (8)	0.030 (2)	
H10A	0.8339	0.4787	0.5842	0.037*	
H10B	0.6737	0.5164	0.4927	0.037*	
C11	0.8301 (13)	0.2914 (9)	0.5380 (7)	0.0221 (16)	
C12	1.0873 (14)	0.1595 (10)	0.3990 (9)	0.0313 (19)	
H12A	1.1489	0.0841	0.4717	0.047*	
H12B	1.1890	0.1780	0.3236	0.047*	
H12C	0.9953	0.1234	0.3756	0.047*	

### Atomic displacement parameters (Å^2^)

**Table:**

	*U*^11^	*U*^22^	*U*^33^	*U*^12^	*U*^13^	*U*^23^
Hg1	0.0304 (3)	0.0247 (3)	0.0247 (3)	−0.00519 (19)	−0.00101 (17)	−0.00514 (17)
Cl1	0.0304 (12)	0.0320 (12)	0.0350 (11)	−0.0028 (10)	−0.0050 (9)	−0.0083 (9)
Cl2	0.0360 (13)	0.0344 (11)	0.0255 (10)	−0.0096 (10)	−0.0038 (9)	−0.0057 (8)
O1	0.027 (3)	0.025 (3)	0.022 (3)	−0.011 (3)	0.017 (2)	−0.009 (2)
O2	0.030 (3)	0.025 (3)	0.028 (3)	−0.006 (3)	0.010 (3)	−0.012 (2)
O3	0.041 (4)	0.025 (3)	0.034 (3)	−0.013 (3)	0.014 (3)	−0.012 (2)
N1	0.027 (4)	0.023 (3)	0.019 (3)	−0.008 (3)	0.002 (3)	−0.009 (3)
C1	0.024 (4)	0.030 (4)	0.021 (4)	−0.007 (4)	0.000 (3)	−0.009 (3)
C2	0.029 (5)	0.039 (5)	0.019 (4)	−0.012 (4)	0.005 (4)	−0.012 (3)
C3	0.018 (4)	0.038 (5)	0.030 (4)	−0.005 (4)	0.003 (3)	−0.019 (4)
C4	0.020 (4)	0.024 (4)	0.022 (4)	−0.001 (3)	0.002 (3)	−0.011 (3)
C5	0.041 (6)	0.025 (4)	0.034 (4)	−0.012 (4)	0.000 (4)	−0.014 (4)
C6	0.034 (5)	0.017 (4)	0.034 (5)	−0.004 (4)	−0.003 (4)	−0.003 (3)
C7	0.035 (5)	0.028 (4)	0.022 (4)	−0.009 (4)	−0.002 (4)	−0.005 (3)
C8	0.021 (4)	0.020 (4)	0.024 (4)	−0.003 (3)	0.003 (3)	−0.009 (3)
C9	0.018 (4)	0.030 (4)	0.020 (3)	−0.006 (3)	−0.001 (3)	−0.009 (3)
C10	0.041 (6)	0.032 (5)	0.018 (4)	−0.015 (4)	0.010 (4)	−0.012 (3)
C11	0.023 (4)	0.024 (4)	0.019 (4)	−0.009 (3)	0.005 (3)	−0.008 (3)
C12	0.023 (5)	0.029 (5)	0.035 (5)	−0.002 (4)	0.011 (4)	−0.018 (4)

### Geometric parameters (Å, º)

**Table:**

Hg1—Cl1	2.340 (2)	C3—C4	1.416 (12)
Hg1—Cl2	2.350 (2)	C3—H3	0.9400
Hg1—N1	2.463 (6)	C4—C9	1.432 (11)
Hg1—O1	2.746 (6)	C4—C5	1.428 (12)
Hg1—O3	2.876 (6)	C5—C6	1.357 (13)
Hg1—Cl1^i^	3.204 (2)	C5—H5	0.9400
O1—C8	1.383 (10)	C6—C7	1.392 (13)
O1—C10	1.396 (10)	C6—H6	0.9400
O2—C11	1.329 (10)	C7—C8	1.361 (12)
O2—C12	1.468 (10)	C7—H7	0.9400
O3—C11	1.195 (10)	C8—C9	1.418 (11)
N1—C1	1.337 (11)	C10—C11	1.502 (12)
N1—C9	1.352 (11)	C10—H10A	0.9800
C1—C2	1.405 (12)	C10—H10B	0.9800
C1—H1	0.9400	C12—H12A	0.9700
C2—C3	1.355 (14)	C12—H12B	0.9700
C2—H2	0.9400	C12—H12C	0.9700
			
Cl1—Hg1—Cl2	153.41 (8)	C3—C4—C5	122.3 (7)
Cl1—Hg1—N1	106.61 (17)	C9—C4—C5	119.5 (7)
Cl2—Hg1—N1	99.30 (17)	C6—C5—C4	119.5 (8)
Cl1—Hg1—O1	105.43 (15)	C6—C5—H5	120.3
Cl2—Hg1—O1	91.75 (15)	C4—C5—H5	120.3
N1—Hg1—O1	62.08 (19)	C5—C6—C7	121.3 (8)
Cl1—Hg1—O3	80.81 (15)	C5—C6—H6	119.4
Cl2—Hg1—O3	92.53 (16)	C7—C6—H6	119.4
N1—Hg1—O3	117.7 (2)	C8—C7—C6	121.2 (8)
O1—Hg1—O3	56.55 (16)	C8—C7—H7	119.4
Cl1—Hg1—Cl1^i^	83.50 (8)	C6—C7—H7	119.4
Cl2—Hg1—Cl1^i^	90.92 (7)	C7—C8—O1	124.5 (7)
N1—Hg1—Cl1^i^	89.63 (16)	C7—C8—C9	120.5 (7)
O1—Hg1—Cl1^i^	151.64 (12)	O1—C8—C9	115.1 (7)
O3—Hg1—Cl1^i^	151.45 (13)	N1—C9—C8	120.7 (7)
C8—O1—C10	116.4 (6)	N1—C9—C4	121.2 (7)
C8—O1—Hg1	115.6 (4)	C8—C9—C4	118.0 (7)
C10—O1—Hg1	128.0 (5)	O1—C10—C11	108.5 (7)
C11—O2—C12	115.3 (7)	O1—C10—H10A	110.0
C11—O3—Hg1	121.0 (5)	C11—C10—H10A	110.0
C1—N1—C9	119.1 (7)	O1—C10—H10B	110.0
C1—N1—Hg1	116.1 (5)	C11—C10—H10B	110.0
C9—N1—Hg1	124.1 (5)	H10A—C10—H10B	108.4
N1—C1—C2	122.4 (8)	O3—C11—O2	124.9 (8)
N1—C1—H1	118.8	O3—C11—C10	125.5 (8)
C2—C1—H1	118.8	O2—C11—C10	109.6 (7)
C3—C2—C1	120.2 (8)	O2—C12—H12A	109.5
C3—C2—H2	119.9	O2—C12—H12B	109.5
C1—C2—H2	119.9	H12A—C12—H12B	109.5
C2—C3—C4	118.8 (8)	O2—C12—H12C	109.5
C2—C3—H3	120.6	H12A—C12—H12C	109.5
C4—C3—H3	120.6	H12B—C12—H12C	109.5
C3—C4—C9	118.2 (7)		
			
Cl1—Hg1—O1—C8	−113.6 (5)	C3—C4—C5—C6	178.0 (9)
Cl2—Hg1—O1—C8	86.9 (5)	C9—C4—C5—C6	−0.6 (13)
N1—Hg1—O1—C8	−12.7 (5)	C4—C5—C6—C7	0.9 (15)
O3—Hg1—O1—C8	178.8 (6)	C5—C6—C7—C8	−1.8 (15)
Cl1^i^—Hg1—O1—C8	−8.3 (7)	C6—C7—C8—O1	−178.0 (8)
Cl1—Hg1—O1—C10	65.7 (7)	C6—C7—C8—C9	2.2 (14)
Cl2—Hg1—O1—C10	−93.8 (7)	C10—O1—C8—C7	13.2 (13)
N1—Hg1—O1—C10	166.6 (8)	Hg1—O1—C8—C7	−167.4 (7)
O3—Hg1—O1—C10	−1.9 (7)	C10—O1—C8—C9	−167.0 (8)
Cl1^i^—Hg1—O1—C10	171.0 (6)	Hg1—O1—C8—C9	12.4 (9)
Cl1—Hg1—O3—C11	−110.2 (7)	C1—N1—C9—C8	178.2 (8)
Cl2—Hg1—O3—C11	95.7 (7)	Hg1—N1—C9—C8	−11.9 (11)
N1—Hg1—O3—C11	−6.1 (8)	C1—N1—C9—C4	−2.1 (11)
O1—Hg1—O3—C11	5.3 (6)	Hg1—N1—C9—C4	167.8 (6)
Cl1^i^—Hg1—O3—C11	−167.7 (5)	C7—C8—C9—N1	177.9 (8)
Cl1—Hg1—N1—C1	−78.3 (6)	O1—C8—C9—N1	−1.9 (11)
Cl2—Hg1—N1—C1	95.7 (6)	C7—C8—C9—C4	−1.8 (12)
O1—Hg1—N1—C1	−177.3 (7)	O1—C8—C9—C4	178.4 (7)
O3—Hg1—N1—C1	−166.5 (5)	C3—C4—C9—N1	2.7 (12)
Cl1^i^—Hg1—N1—C1	4.8 (6)	C5—C4—C9—N1	−178.7 (8)
Cl1—Hg1—N1—C9	111.5 (6)	C3—C4—C9—C8	−177.6 (8)
Cl2—Hg1—N1—C9	−74.5 (6)	C5—C4—C9—C8	1.0 (11)
O1—Hg1—N1—C9	12.5 (6)	C8—O1—C10—C11	178.6 (7)
O3—Hg1—N1—C9	23.4 (7)	Hg1—O1—C10—C11	−0.7 (11)
Cl1^i^—Hg1—N1—C9	−165.4 (6)	Hg1—O3—C11—O2	173.0 (6)
C9—N1—C1—C2	−1.0 (12)	Hg1—O3—C11—C10	−8.8 (12)
Hg1—N1—C1—C2	−171.7 (7)	C12—O2—C11—O3	−1.6 (12)
N1—C1—C2—C3	3.6 (14)	C12—O2—C11—C10	179.9 (7)
C1—C2—C3—C4	−2.9 (13)	O1—C10—C11—O3	6.4 (13)
C2—C3—C4—C9	−0.1 (12)	O1—C10—C11—O2	−175.1 (7)
C2—C3—C4—C5	−178.7 (9)		

Symmetry code: (i) −*x*+1, −*y*, −*z*+2.
